# QT Prolongation After His Bundle Pacing: What Is the Mechanism?

**DOI:** 10.1016/j.jaccas.2021.11.015

**Published:** 2022-02-16

**Authors:** Donald Mehlhorn, Jessica Bass, Kumar Narayan, Dinesh Sharma

**Affiliations:** a Naples Heart Institute, NCH Healthcare System, Naples, Florida, USA; b Medicover Hospitals, Hyderabad, India

**Keywords:** acute heart failure, cardiac pacemaker, cardiomyopathy, ECG, electrocardiography, HBP, His bundle pacing, LBBB, left bundle branch block, RV, right ventricle

## Abstract

An 82-year-old woman with nonischemic cardiomyopathy underwent cardiac resynchronization therapy by the use of His bundle pacing. After the procedure, the patient had repolarization abnormality with severely prolonged QTc and anterior inferior T-wave inversions, likely resulting from memory T waves associated with the correction of long-standing left bundle branch block. These changes could be potentially arrhythmogenic. (**Level of Difficulty: Intermediate.**)

## Graphical abstract

**Figure:**
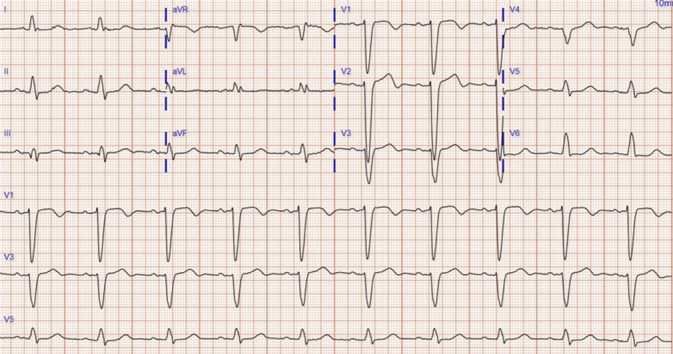

Prolonged QTc and memory T-wave changes may occur secondary to significant alterations in depolarization. Previously, severe QT prolongation due to LV pacing, which could be corrected by His bundle pacing (HBP) has been reported.^1^ Paradoxically, memory T waves after HBP have also been reported.^2^ Here, we report a case of repolarization abnormalities and prolonged QTc that occurred with His bundle pacing in a patient with prior wide QRS.

**Learning Objectives**

• To be cognizant of the phenomenon of severe QTc prolongation secondary to normalization of QRS with His bundle pacing.
• To understand the electrocardiographic changes that are associated with cardiac or T-wave memory.
• To understand the importance of considering cardiac or T-wave memory as an explanation before performing potentially unnecessary therapy or treatments.

## History of Presentation

An 82-year-old woman was admitted because of congestive heart failure exacerbation. On examination, she was afebrile, with heart rate 74 beats/min, respiratory rate 18/min, blood pressure 153/61 mm Hg, and oxygen saturation 92% on room air. She was morbidly obese with positive jugular venous distension and lower extremity pitting edema. There were no murmurs on auscultation, and her lungs were clear.

## Medical History

She had a history of paroxysmal atrial fibrillation, was taking amiodarone and metoprolol and anticoagulant therapy with coumadin, had a left bundle branch block (LBBB), and heart failure with reduced ejection fraction with a last known ejection fraction of 20% to 25% for the past 1 year, diabetes, and chronic kidney disease stage III.

## Investigations

**Figure 1:**
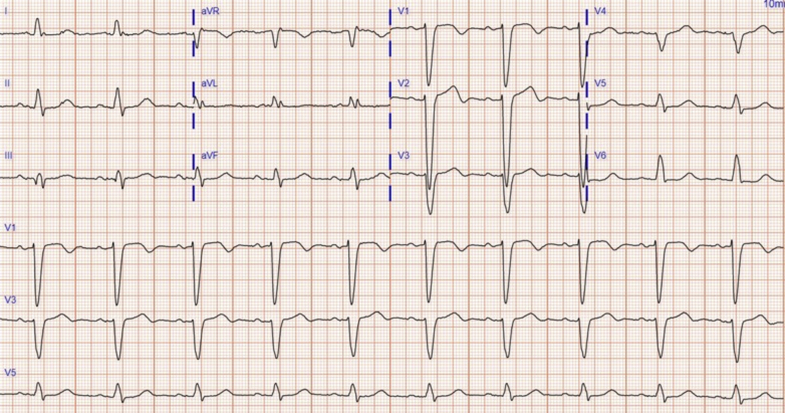
Baseline Electrocardiogram With Left Bundle Branch Block Electrocardiogram showing QTc 567 ms (QRS 168 ms).

Her chest x-ray was remarkable for prominent pulmonary vasculature and interstitial markings. An electrocardiography (ECG) on admission revealed LBBB (Figure 1). A repeated transthoracic echocardiogram confirmed depressed ejection fraction at 20% to 25%.

## Management

Although our patient had been receiving guideline-directed medical therapy for >1 year, she unfortunately did not experience any improvement in her symptoms or ejection fraction. During her admission for congestive heart failure exacerbation the patient received intravenous diuresis with the plan to place a cardiac resynchronization therapy defibrillator after she was euvolemic.

**Figure 2:**
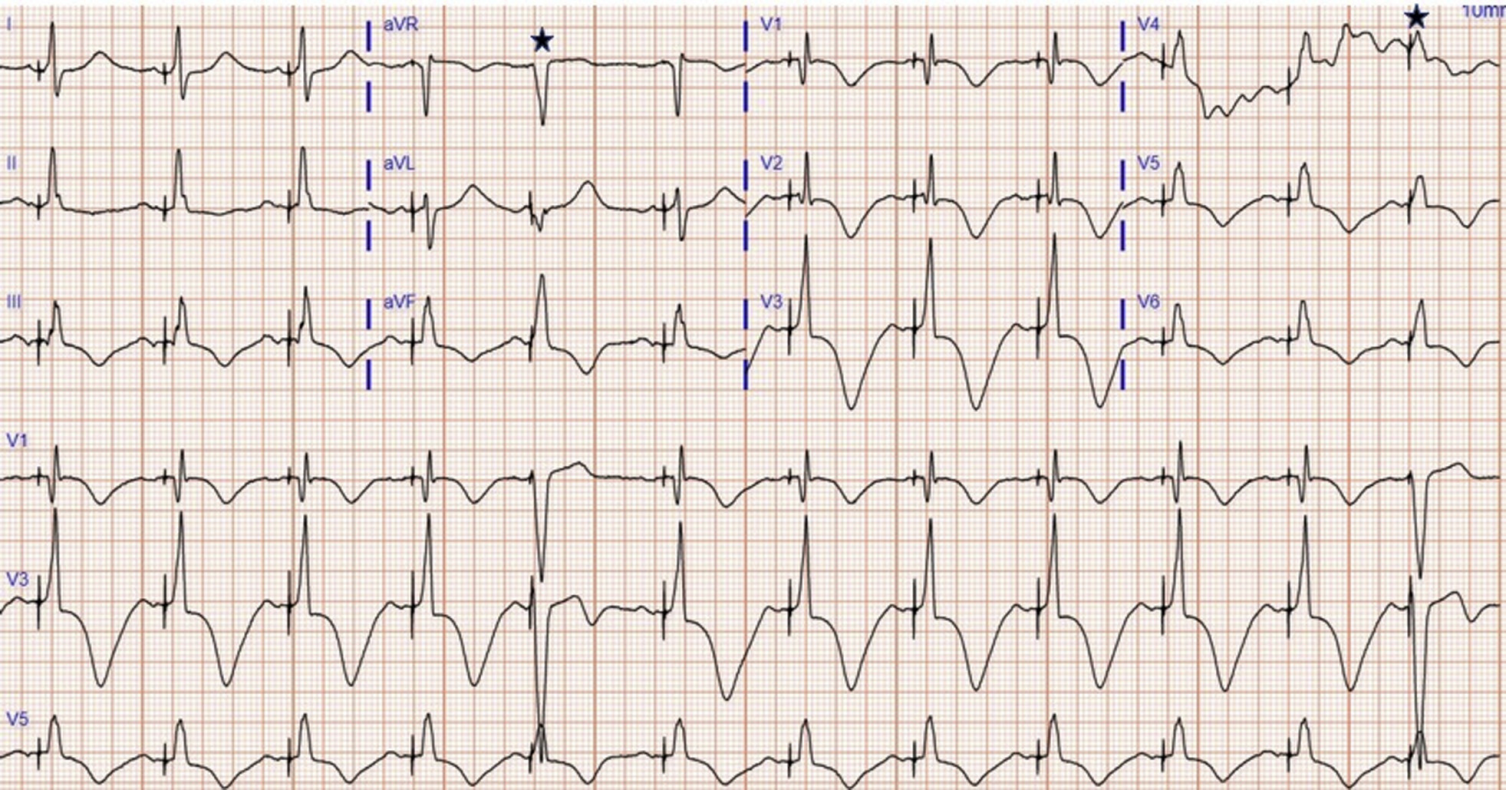
Postprocedure Electrocardiogram With His Bundle Pacing and Normalized QRS ∗Intermittent right ventricle septal capture. Right ventricle septal capture QTc 551 ms (QRS 137 ms) and during His bundle pacing QTc 600 ms (QRS 90 ms). **(A)** Electrocardiogram on postoperative day 2 showing QTc 533 ms. **(B)** Electrocardiogram on postoperative day 32 showing QTc 506 ms.

During the cardiac resynchronization therapy defibrillator procedure, the cannulated posterolateral vein had an unacceptably high capture threshold. Hence, as an alternative, an HBP lead was implanted with complete LBBB correction and normalization of the QRS complex. The threshold at implantation was 0.5 V at 0.4 ms, and output was set to 3.0 V at 0.4 ms. Next day the ECG revealed His bundle capture and intermittent right ventricle (RV) septal capture (Figure 2). In comparison with RV septal capture, HB capture had severely prolonged QTc at 600 ms. A basic metabolic panel revealed electrolytes within normal limits. Because of the intermittent capture, the His capture threshold was rechecked and found to be 1.5 V at 1.0 ms pulse width; hence, the output was changed to 3.0V at 1.0 ms.

**Figure 3:**
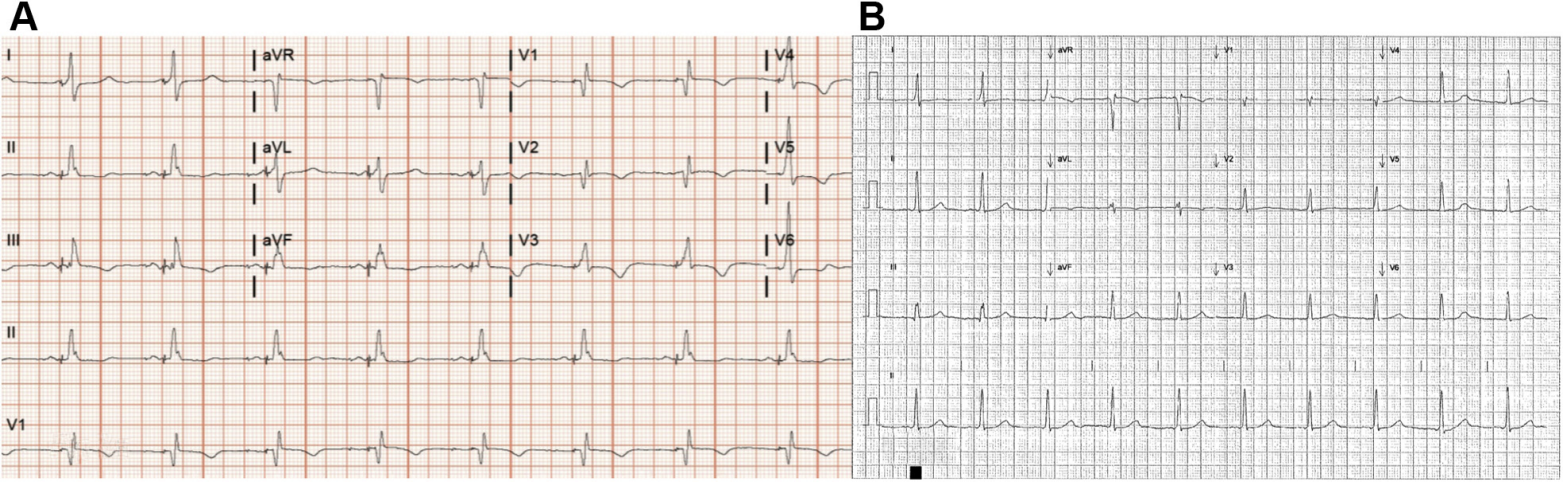
Postoperative Electrocardiograms **(A)** Electrocardiogram on postoperative day 2 showing QTc 533 ms. **(B)** Electrocardiogram on postoperative day 32 showing QTc 506 ms.

In view of the severe QT prolongation, the patient was monitored in the hospital for 2 additional days, during which the QTc gradually reduced to 533 ms (Figure 3A). The patient’s condition was stable during this time, with no acute events that warranted intervention. She was discharged to cardiac rehabilitation with improvement in her QTc on follow-up (Figure 3B)

## Differential Diagnosis

The differential diagnosis of prolonged QTc with normalization of QRS includes memory T waves, electrolyte derangement, potassium channel blocking agents such as amiodarone, left ventricle strain, or ischemia. The memory T waves can be seen in conditions such as correction of LBBB, ventricular tachycardia, pre-excitation, and sustained RV pacing.

## Discussion

Cardiac memory refers to persistent T-wave changes that develop after a period of abnormal ventricular activation (wide QRS complexes), once ventricular depolarization changes.

In this phenomenon, the T-wave remembers and follows the direction of the original wide QRS complexes even though normal ventricular activation has been restored. In the present case, inverted T waves in inferior leads are unusual, and we speculate that the potential reason may be that the overall major change in ventricular depolarization due to HBP may have resulted in repolarization changes beyond just memory T waves. Cardiac memory is not pathologic and can persist up to weeks after the heart’s normal conduction is restored.^3^

The patient was in a potentially arrhythmogenic state immediately after QRS normalization because of severely prolonged QTc approximately 600 ms. It was likely due to a combination of memory T waves, amiodarone, and underlying cardiomyopathy. Improvement in QTc was observed during intermittent RV capture (Figure 2), which supports the diagnosis of memory T waves. Increase in HBP output led to consistent His bundle capture. After 2 days of inpatient observation, QTc improved.

Patients with cardiomyopathy and wide QRS could experience significant QT prolongation with potential for an arrhythmogenic state immediately after normalization of the QRS because of the memory phenomenon. At least, overnight observation under telemetry with QTc monitoring should be considered in such cases.

## Follow-Up

The patient has had no further admissions or adverse events since her discharge from the hospital. A 1-month follow-up echocardiogram revealed that ejection fraction had normalized.

## Conclusions

This case demonstrates the occurrence of significant repolarization abnormality after normalization of QRS with His bundle pacing. Clinicians should be aware of this phenomenon, which may require additional monitoring.

## Funding Support and Author Disclosures

The authors have reported that they have no relationships relevant to the contents of this paper to disclose.

## References

[1] Lador A., Valderrábano M. (2020). QT interval prolongation and torsade de pointes induced by left ventricular pacing rescued by His bundle pacing. HeartRhythm Case Rep.

[2] Vijayaraman P., Dandamudi G., Miller J.M. (2014). Paradoxical cardiac memory during permanent His bundle pacing. J Cardiovasc Electrophysiol.

[3] Shvilkin A., Huang H.D., Josephson M.E. (2015). Cardiac memory: diagnostic tool in the making. Circ Arrhythm Electrophysiol.

